# Tiragolumab and TIGIT: pioneering the next era of cancer immunotherapy

**DOI:** 10.3389/fphar.2025.1568664

**Published:** 2025-06-11

**Authors:** Kosar Ghasemi

**Affiliations:** ^1^ Department of Pharmacology and Toxicology, School of Pharmacy, Jundishapur University of Medical Sciences, Ahvaz, Iran; ^2^ Cellular and Molecular Research Center, Jundishapur University of Medical Sciences, Ahvaz, Iran

**Keywords:** tiragolumab, TIGIT, cancer immunotherapy, immune checkpoint inhibitor 19, cancer

## Abstract

Tiragolumab, a monoclonal antibody (mAb) targeting T cell immunoreceptor with Ig and ITIM domains (TIGIT), represents a novel approach in cancer immunotherapy. TIGIT, an immunological checkpoint receptor, suppresses T cell activation and promotes immune evasion in various cancers. By inhibiting TIGIT, Tiragolumab enhances T cell-mediated anti-tumor immunity, particularly when combined with programmed cell death-1 (PD-1) and programmed death-ligand 1 (PD-L1) inhibitors. This synergy arises from complementary mechanisms, where TIGIT blockade reduces CD155-mediated suppression, amplifying PD-1/PD-L1-driven T cell activation. Phase II and III trials, including the CITYSCAPE trial for non-small cell lung cancer (NSCLC), have shown improved objective response rates (37% vs. 21% with PD-L1 inhibitor monotherapy) and progression-free survival (PFS), with manageable adverse effects. However, the potential of other checkpoint inhibitors, such as Lymphocyte Activation Gene 3 (LAG3), T-cell immunoglobulin and mucin domain-3 (TIM-3), or cytotoxic T-lymphocyte-associated protein 4 (CTLA-4), remains underexplored compared to TIGIT. This review summarizes TIGIT’s molecular mechanisms, preclinical and clinical data, and limitations, including resistance mechanisms (e.g., upregulation of alternative checkpoints), biomarker development, and the need for broader investigation into alternative inhibitors to optimize combination therapies for personalized, durable cancer treatment.

## 1 Introduction

Immunotherapy has revolutionized cancer treatment, particularly with the advent of immune checkpoint inhibitors (ICIs) that target programmed cell death protein 1 (PD-1), programmed death-ligand 1 (PD-L1), and cytotoxic T-lymphocyte-associated protein 4 (CTLA-4) (Shiravand et al., 2022; Cai et al., 2024; Zhu et al., 2024; Cheng et al., 2024). These medications have demonstrated enduring and promising clinical outcomes in several cancers. Clinical studies indicate that the remission rate for patients treated with CTLA-4 inhibitors is approximately 15%, whereas the remission rate for PD-1/PD-L1 inhibitors rarely exceeds 40% (Wolchok et al., 2017). Moreover, suboptimal clinical target response rates and the risk of inducing autoimmune disorders remain the primary drawbacks of ICIs (Kumar et al., 2017; De Velasco et al., 2017; De Silva et al., 2021). Additionally, the evidence indicates that some patients remain resistant to ICIs, despite effective tumor suppression, resulting in certain patients not benefiting from ICI therapy (Lao et al., 2022; O'Donnell et al., 2017; Cui et al., 2024).

T cell immunoreceptor with Ig and ITIM domains (TIGIT) is an essential co-inhibitory receptor found on T cells and natural killer (NK) cells, playing a vital role in immune evasion (Sun et al., 2024a; Ghasemi, 2024; Ke, 2024). It inhibits the function of effector T cells and facilitates the tumor microenvironment (TME) (Harjunpää and Guillerey, 2020). TIGIT interacts with its ligands, CD155 (PVR) and CD112 (Nectin-2), which are often overexpressed on tumor cells and antigen-presenting cells (APCs) within the TME (Gorvel and Olive, 2020; Jo et al., 2024). This interaction leads to reduced T cell activation, diminished cytokine production, and increased recruitment of immunosuppressive regulatory T cells (Tregs) (Kurtulus et al., 2015). TIGIT signaling in Tregs has been shown to influence the phenotype of CD8^+^ T cells, mainly suppressing antitumor immunity via Tregs rather than CD8^+^ T cells (Lang et al., 2024). Additionally, TIGIT+ Tregs enhanced the expression of the coinhibitory receptor T-cell immunoglobulin and mucin-domain containing-3 (TIM-3) within tumor tissue (Joller et al., 2024). Consequently, TIM-3 and TIGIT collaborated to block antitumor immune responses (Kurtulus et al., 2015). Thus, inhibiting the TIGIT pathway has emerged as a compelling approach for revitalizing anti-tumor immunity (Jiang F. et al., 2024).

Tiragolumab, a monoclonal antibody (mAb) targeting TIGIT, has demonstrated favorable outcomes in preclinical and early clinical investigations (Rotte et al., 2021; Mu and Guan, 2024). Blocking TIGIT with Tiragolumab restores T cell effector capabilities and enhances NK cell activity (Florou and Garrido-Laguna, 2022; Eichberger et al., 2022). Its effectiveness is significantly enhanced when combined with PD-1/PD-L1 inhibitors, resulting in a dual blockade that targets multiple pathways of immune suppression (Kim et al., 2023). Most adverse events (AEs) were mild, with immune-mediated AEs occurring in 17% (phase 1a) and 59% (phase 1b). While no responses were confirmed in phase 1a, phase 1b demonstrated a 46% response rate in non-small cell lung cancer (NSCLC) and 28% in esophageal cancer (EC), maintaining consistent safety when combined with atezolizumab (Kim et al., 2023; Rousseau et al., 2023).

This review discusses the TIGIT pathway, the mechanisms of action of Tiragolumab, and its clinical advancements. It also addresses challenges such as identifying predictive biomarkers, understanding resistance mechanisms, and optimizing combination therapies. The article emphasizes the significance of Tiragolumab in shaping the future of cancer immunotherapy.

## 2 Immunosuppressive tumor microenvironment and inhibitory molecules

The TME orchestrates various mechanisms in solid tumors to suppress anti-tumor immune responses, thus creating a supportive environment for cancer progression and immune evasion (Giraldo et al., 2019; Mortezaee, 2020). The interactions among tumor cells, immune cells, stromal components, and signaling molecules shape the immunosuppressive properties of the tumor site. A key feature of the TME is its ability to inhibit effector immune cells, such as CD8^+^ cytotoxic T lymphocytes (CTLs) and NK cells (Xie et al., 2021; Li et al., 2020).

TME primarily induces immune suppression via immune checkpoints (Toor, 2020). For example, PD-L1 in tumor cells interacts with the PD-1 receptor on the surface of T cells, leading to T cell exhaustion and reduced cytotoxic efficacy (Zhang et al., 2020). Similarly, CTLA-4 obstructs T-cell activation by competing with the co-stimulatory receptor CD28 for binding to the CD80 and CD86 co-stimulatory molecules located on APCs (McCoy and Le Gros, 1999). This competitive inhibition impedes the full activation of T cells, promoting tumor progression. Alongside the immune checkpoints, the TME attracts several immunosuppressive cell types, such as tumor-associated macrophages (TAMs), cancer-associated fibroblasts (CAFs), tumor-associated neutrophils (TANs), Tregs, and myeloid-derived suppressor cells (MDSCs) (Haist et al., 2021; Lindau et al., 2013; Li et al., 2021; Gunaydin, 2021). These cells release anti-inflammatory cytokines, including interleukin-10 (IL-10), transforming growth factor-beta (TGF-β), and other protumor mediators that inhibit effector immune responses and foster a tolerogenic and tumor-supportive milieu (Rajani et al., 2019). For instance, recent reports indicate that CAFs interact with cancer and immune cells by secreting cytokines and vesicles, modifying the extracellular matrix (ECM), and modulating the immune response (Pei et al., 2023). These interactions often create an immunosuppressive environment that negatively impacts treatment effectiveness. Recent investigations into CAF heterogeneity have identified various subtypes with distinct activities in tumor growth, offering new opportunities for combination immunotherapy by targeting specific CAF functions and predicting immune response through CAF-related biomarkers, thereby enhancing the effectiveness of immunotherapies for resistant malignancies (Pei et al., 2023).

**FIGURE 1:**
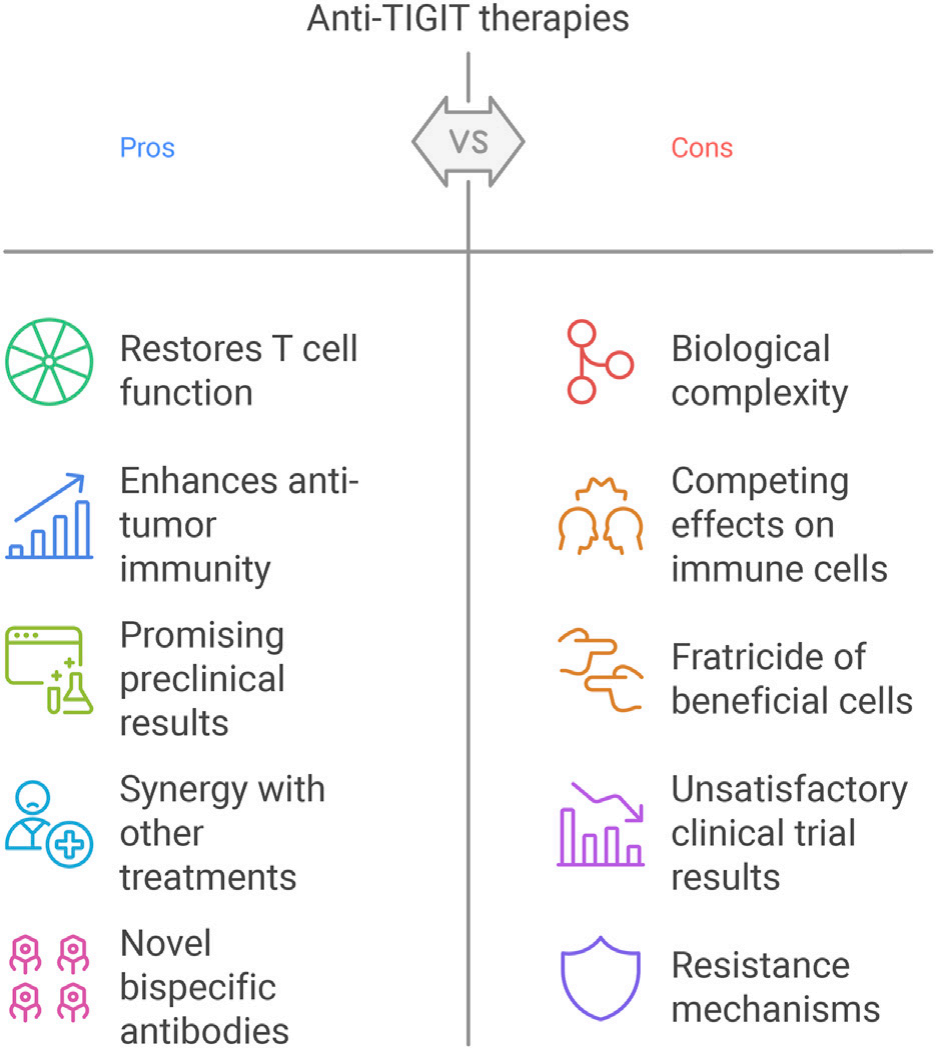
Mechanisms of immune suppression and tumor progression in the TME. The TME consists of tumor, stromal, and immune cells, which interact to create an immunosuppressive milieu. MDSCs, TAMs, CAFs, and Tregs contribute to this immunosuppressive environment by producing anti-inflammatory cytokines, such as TGF-β and IL-10, as well as enzymes like IDO and arginase. These factors deplete essential nutrients like tryptophan and arginine and remodel the ECM, facilitating tumor growth and invasion. ROS further contributes to immune dysfunction, while tumor and immune cells express immune checkpoint molecules, including PD-L1 and CTLA-4. These checkpoints interact with their respective receptors on CD8^+^ T cells, leading to T-cell exhaustion or inactivation. The shift in macrophage polarization from pro-inflammatory (M1) to anti-inflammatory (M2) phenotypes and the suppression of NK cell activity also impair anti-tumor immunity. The interplay of these factors results in enhanced tumor cell survival, angiogenesis, and metastasis, underscoring the complexity and therapeutic challenges of targeting the TME. MDSC, myeloid-derived suppressor cell; TAM, tumor-associated macrophage; CAF, cancer-associated fibroblast; Treg, regulatory T cell; TGF-β, transforming growth factor-beta; IL-10, interleukin-10; PD-L1, programmed death-ligand 1; CTLA-4, cytotoxic T-lymphocyte-associated protein 4; ROS, reactive oxygen species; ECM, extracellular matrix.

Metabolic suppression significantly affects the immunosuppressive properties of TME components. Enzymes such as indoleamine 2,3-dioxygenase (IDO) and arginase are elevated in the TME, leading to the depletion of essential nutrients like tryptophan and arginine, critical for T cell proliferation and function (Wang et al., 2024; Molinier‐Frenkel and Castellano, 2017). Reactive oxygen species (ROS) generated by MDSCs further inhibit T cell activity, while IDO-mediated depletion of tryptophan directly reduces T cell survival and effector function. Together, these substances create a metabolically detrimental environment for immune cells, intensifying immune suppression (Figure 1).

These inhibitory mechanisms create significant obstacles to effective immune responses in the TME. Therapies targeting these pathways, such as ICIs (anti-PD-1 and anti-CTLA-4 antibodies), have shown promise in restoring T cell function and enhancing anti-tumor immunity (Marei et al., 2023; Kon and Benhar, 2019). However, several obstacles, such as tumor heterogeneity, the expression of inhibitory molecules and mediators, immune evasion mechanisms, and resistance to checkpoint blockade, highlight the complexity of the TME (El-Sayes et al., 2021; Bhat et al., 2024). Tumors may adapt by enhancing alternative suppressive pathways or increasing the recruitment of immunosuppressive cells, necessitating combination therapies that address multiple aspects of immune suppression (Stewart and Smyth, 2011; Seliger and Massa, 2021).

The immunosuppressive tumor milieu, taken together, comprises a complex network that obstructs anti-tumor immunity through inhibitory substances, immune checkpoint mechanisms, and metabolic suppression (Wegiel et al., 2018; Labani-Motlagh et al., 2020). Ongoing studies on these systems may provide more effective therapies to overcome immune resistance, thereby improving cancer clinical outcomes.

## 3 Role of TIGIT in the tumor microenvironment and cancer immunopathology

**FIGURE 2:**
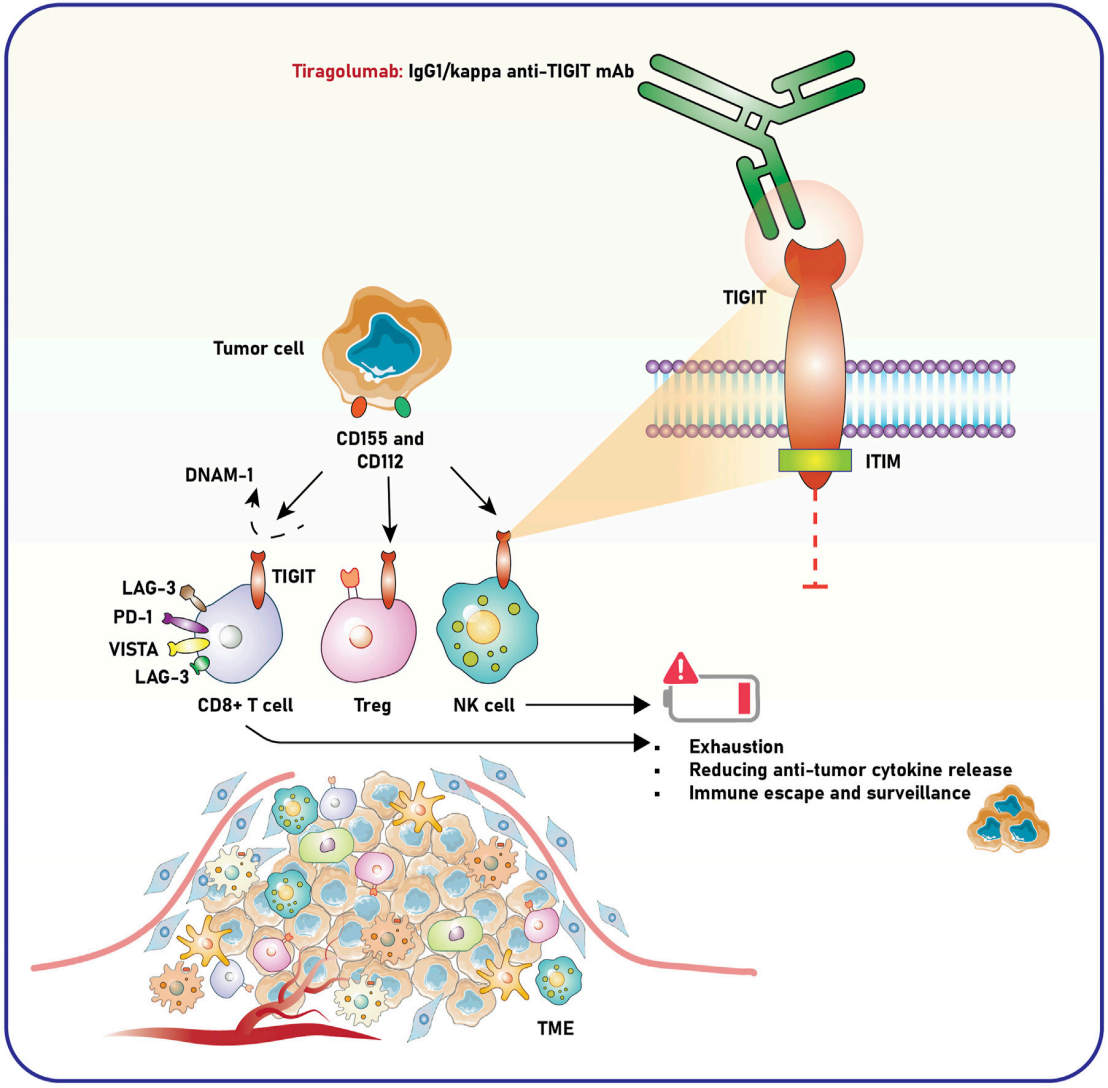
Role of TIGIT and anti-TIGIT monoclonal antibody in modulating immune responses within the TME. Tumor cells express ligands CD155 and CD112, which interact with immune checkpoint receptors such as TIGIT on CD8^+^ T cells, Tregs, and NK cells. This interaction suppresses immune activation, leading to T-cell exhaustion, decreased release of anti-tumor cytokines, and impaired NK cell-mediated tumor surveillance, ultimately facilitating immune escape and tumor progression. Co-inhibitory molecules, including LAG-3, PD-1, and VISTA, further enhance this immunosuppressive state. Tiragolumab, an IgG1/kappa anti-TIGIT monoclonal antibody, blocks TIGIT signaling by preventing its engagement with CD155 and CD112. This blockade reduces immune suppression and restores the activity of CD8^+^ T cells and NK cells, promoting anti-tumor immunity. The intracellular ITIM domain of TIGIT mediates its inhibitory signaling, contributing to immune cell dysfunction in the TME. The schematic highlights how targeting TIGIT with tiragolumab may counteract immune exhaustion and enhance anti-tumor responses. TIGIT, T-cell immunoreceptor with Ig and ITIM domains; ITIM, immunoreceptor tyrosine-based inhibitory motif; LAG-3, lymphocyte-activation gene 3; PD-1, programmed death-1; VISTA, V-domain Ig suppressor of T-cell activation; DNAM-1, DNAX accessory molecule-1; NK cell, natural killer cell; Treg, regulatory T cell; TME, tumor microenvironment.

TIGIT is an inhibitory immune checkpoint receptor expressed by several immune cells, including T cells, NK cells, and Tregs (Harjunpää and Guillerey, 2020). It has a critical role in the tumor milieu, where its interactions with ligands, including CD155 and CD112, enable immune evasion and advance cancer development (Freed-Pastor et al., 2021). Accordingly, targeting TIGIT is considered a promising therapeutic approach in cancer immunopathology. TIGIT is increasingly expressed on exhausted and dysfunctional effector T cells and NK cells within the TME, primarily due to prolonged antigen stimulation and the immunosuppressive environment created by tumor cells (Hossain et al., 2021; Zhang et al., 2018). In contrast to CTLA-4 and PD-1 checkpoint molecules, TIGIT is associated with NK cell exhaustion in mice with tumors and patients with colon cancer (Sun et al., 2024a). Blocking TIGIT prevents NK cell exhaustion and boosts NK cell-mediated tumor immunity in various tumor-bearing mouse models. Additionally, inhibiting TIGIT enhances tumor-specific T cell immunity through an NK cell-dependent process, improves therapeutic results with anti-PD-L1 antibodies, and extends memory immunity during tumor rechallenge in animals (Zhang et al., 2018) (Figure 2).

TIGIT binds to CD155, an overexpressed ligand in tumor and stromal cells within the TME, transmitting inhibitory signals through its immunoreceptor tyrosine-based inhibitory motif (ITIM). This mechanism reduces the cytotoxic activity of T and NK cells, thereby impairing the immune system’s ability to eliminate tumor cells (Jiang S. et al., 2024; Annese et al., 2022). TIGIT enhances the suppressive capabilities of Tregs, which are crucial for maintaining the immunosuppressive TME. A study underscored the significance of TIGIT, a coinhibitory receptor, in modulating Th1 Tregs—a subgroup of Tregs characterized by diminished suppressor activity and increased proinflammatory cytokine production, often linked to autoimmune disorders (Lucca et al., 2019). IL-12 activation stimulates Th1 Tregs by hyperactivating the Akt pathway, resulting in their dysfunction. TIGIT signaling counteracts this process by inhibiting interferon-gamma (IFN-γ) production, reducing T-bet expression, and restoring Treg suppressor function, supported by decreased Akt activity and enhanced FoxO1 nuclear localization. TIGIT activation in cancer may address Treg deficiencies and reduce inflammatory cytokine production (Lucca et al., 2019). The outcomes designate TIGIT as a vital regulator of Treg stability and a potential therapeutic target for cancer and autoimmune diseases.

TIGIT has a dual function—impeding effector cells while enhancing suppressor cells—thereby strengthening the immunosuppressive networks that allow tumors to evade immune surveillance (Jiang S. et al., 2024). TIGIT competes with costimulatory receptors like DNAX accessory molecule-1 (DNAM-), also known as CD226, for binding to CD155 (Shibuya and Shibuya, 2021). TIGIT efficiently obstructs costimulatory signals essential for activating effector T cells and NK cells by outcompeting CD226, thus diminishing anti-tumor immunity. The activating immunoreceptor DNAM-1 critically regulates Treg cell function by modulating TIGIT signaling. Treg cells expressing the transcription factor Foxp3 are vital for maintaining immune tolerance but may become dysfunctional under inflammatory conditions. The absence of DNAM-1 enhances Tregs’ ability to mitigate graft-versus-host disease compared to wild-type Tregs. DNAM-1 competes with TIGIT for binding to their shared ligand CD155, thereby attenuating TIGIT signaling without necessitating DNAM-1’s intracellular signaling. DNAM-1 deficiency strengthens TIGIT signaling, suppressing the Akt-mTORC1 pathway, stabilizing Foxp3 expression, and preserving Treg function in inflammatory environments (Sato et al., 2021). Therefore, DNAM-1 can serve as a molecular target to improve Treg-mediated immune regulation in inflammatory diseases.

TIGIT also plays a role in tumor immunopathology by inducing T-cell exhaustion, as evidenced by reduced proliferation, decreased cytokine production, and diminished antitumor functionality (Catakovic et al., 2017). Depleted T cells in the TME co-express several inhibitory receptors, including PD-1, TIM-3, V-domain immunoglobulin suppressor of T-cell activation (VISTA), and lymphocyte activation gene 3 (LAG-3), along with TIGIT, indicating that TIGIT works with other checkpoints to maintain T-cell dysfunction (Huang et al., 2022). TIGIT+ Tregs in the TME are linked to poor clinical outcomes for patients with various malignancies, including NSCLC, melanoma, and colorectal cancer (CRC), emphasizing their role in immune suppression (Fourcade et al., 2018; Peng et al., 2022; Zhou et al., 2021). It has been reported that TIGIT and PD-1 are upregulated in CRC with mismatch repair deficiency, with higher expression observed in cancer tissues than in adjacent normal mucosa. Upregulated levels of TIGIT and PD-1 are associated with advanced TNM stages and improved disease-free survival (DFS), although high PD-1 expression correlates with poorer overall survival (OS). These findings highlight the potential roles of TIGIT and PD-1 in CRC progression and patient prognosis (Zhou et al., 2021).

TIGIT attenuates NK-cell-mediated tumor immunity by reducing cytotoxicity and impeding cytokine production, such as IFN-γ (Jiang P. et al., 2024). This suppression further limits the immune system’s ability to recognize tumor cells that evade identification by T cells, particularly in “cold” tumors characterized by an absence of tumor-infiltrating lymphocytes (TILs) (Liu and Sun, 2021). TIGIT is a compelling therapeutic target due to its essential role in immune evasion. Clinical research is underway for mAbs that selectively target TIGIT, used as both monotherapies and combined with other ICIs, including anti-PD-1/PD-L1 antibodies (Ahn et al., 2020; Grapin et al., 2019). Initial clinical trial results suggest that TIGIT inhibition may enhance T cell and NK cell function, restore anti-tumor immunity, and offer synergistic benefits with existing immunotherapies.

Poliovirus receptor (PVR, CD155) and CD112 are ligands expressed on tumor cells that interact with immune receptors, influencing immune suppression and activation (Wu et al., 2024; Murakami and Ganguly, 2024). A study used mathematical modeling to explore the effects of inhibiting TIGIT, PVRIG, PVR, or their combinations on receptor engagement in immunological synapses (IS) between T-cells and tumor cells (Demin et al., 2024). Results showed that TIGIT inhibition did not affect DNAM1 engagement, while PVRIG inhibition increased DNAM1 binding by 1.3-fold. Blocking PVR increased total DNAM1 and moderately enhanced DNAM1 binding while disrupting TIGIT and CD96 interactions. The combination of PVR and PVRIG blockade had the most significant effect, doubling DNAM1 binding and disrupting multiple immune receptor interactions. These findings suggest that targeting PVR (alone or with PVRIG) may be a more effective strategy for enhancing immune activation than TIGIT inhibition. This could explain why TIGIT inhibitors like Tiragolumab have shown limited success in clinical trials (Demin et al., 2024) (Figure 2).

TIGIT has emerged as a promising immunotherapy target, yet phase III trials with Tiragolumab (anti-TIGIT) and anti-PD-L1 revealed disappointing results (Hasan, 2023). This was linked to the Fc-active domain of anti-TIGIT antibodies, which binds to Fc receptors on NK cells, promoting antibody-dependent cellular cytotoxicity (ADCC) to clear TIGIT^+^ exhausted T cells and Tregs while also causing fratricide in activated TIGIT^+^ NK cells. This fratricide depletes NK cells and impairs their anti-tumor activity. A study found that TIGIT expression on activated NK cells enhances their anti-tumor function; however, chronic TIGIT engagement by its ligand PVR induces dysfunction. Fc-silent anti-TIGIT restored NK cell activity, whereas Fc-active anti-TIGIT reduced NK-mediated killing by depleting TIGIT^+^ NK cells. To overcome this, NK cells were expanded using the PM21-particle method and genetically engineered with CRISPR/Cas-9 to create TIGIT knockout (KO) PM21-NK cells. These TIGIT KO cells improved metabolic fitness, maintained cytotoxicity, and were protected from fratricide induced by Fc-active anti-TIGIT. These findings suggest that Fc-active anti-TIGIT can impair NK cell function through fratricide, but combining it with fratricide-resistant TIGIT KO PM21-NK cells can enhance therapeutic outcomes (Hasan, 2023).

## 4 Targeting TIGIT with tiragolumab in cancer therapies

Effector T cell activity was improved with checkpoint blockage, resulting in long-term remission in a fraction of cancer patients across distinct cancer types (Zebley et al., 2024). Although TIGIT, a checkpoint receptor, was implicated in triggering T cell fatigue within malignancies, its involvement in NK cell failure remained unclear (Harjunpää and Guillerey, 2020; Khan et al., 2020). An investigation showed that TIGIT, rather than CTLA-4 or PD-1, was a remarkable factor in NK cell exhaustion in tumor-bearing mice and colon cancer patients. Blockade of TIGIT inhibited NK cell depletion and promoted NK cell-mediated tumor immunity across many mouse tumor types. Additionally, TIGIT inhibition produced strong tumor-specific T cell immunity reliant on NK cells, increased the effectiveness of PD-L1 antibody treatment, and created durable memory immunity in tumor re-challenge animals. These results identified TIGIT as a crucial and hitherto overlooked checkpoint in NK cells, indicating that targeting TIGIT alone or in conjunction with other checkpoint receptors may offer a feasible treatment strategy for cancer (Zhang et al., 2018).

As discussed, Tiragolumab, a fully human IgG1/kappa anti-TIGIT mAb, has been shown to inhibit the interaction between TIGIT and CD155 (Florou and Garrido-Laguna, 2022) (Figure 2). Pharmacokinetics (PK) data were meticulously analyzed from the phase 1a/1b GO30103 trial, which investigated the administration of Tiragolumab either sequentially every 3 weeks (Q3W) at doses ranging from 2 to 1,200 mg combined with atezolizumab at 1,200 mg, sequentially every 4 weeks (Q4W) at 840 mg Tiragolumab followed by 1,680 mg atezolizumab, or as a Q4W co-infusion of both agents. Serum samples collected at multiple time points revealed that Tiragolumab exhibited a biphasic serum PK profile, characterized by a rapid distribution phase followed by a slower elimination phase. Dose-proportional increases in Tiragolumab exposure were observed at doses equal to or exceeding 100 mg when administered alone or alongside atezolizumab. For doses ranging from 2 to 1,200 mg (cycle 1), the geometric mean (GM) and coefficient of variation (CV%) for serum Tiragolumab C_max_ ranged from 0.682 to 270 μg/mL (18.6%–36.5%), whereas Cmin ranged from 0.0125 to 75.3 μg/mL (0.0%–24.2%). The GM systemic exposure (AUC0-21) varied from 310 to 2,670 µg·day/mL, with interindividual variability in AUC0-21 between 20.5% and 43.9%. Treatment-emergent antidrug antibodies (ADA) were detected in 1.9% of patients (4/207), with each incidence occurring simultaneously. No drug-drug interactions or significant immunogenicity issues were identified between Tiragolumab and atezolizumab. Furthermore, no substantial differences in Tiragolumab or atezolizumab exposure were observed between the Q4W co-infusion and sequential dosing cohorts, thereby supporting the favorable PK profile of Tiragolumab in combination therapy (Garralda et al., 2024).

The phase 1a/1b clinical trial designated GO30103 meticulously assessed the efficacy of Tiragolumab, both as a standalone treatment and in conjunction with atezolizumab, in patients afflicted with advanced solid tumors. No dose-limiting toxicities were discerned, leading to a recommendation to maintain a dosing regimen set at 600 mg administered every 3 weeks. The adverse events reported were predominantly mild, categorized as grade 1 or 2, with fatigue and pruritus recognized as the most frequently documented side effects. Preliminary evidence of antitumor activity was observed, disclosing objective response rates of 46% in NSCLC and 28% in esophageal cancer. The combination therapy exhibited a consistently favorable safety profile and potential efficacy, warranting further investigation (Kim et al., 2023).

The impact of alternative radiation (RT) fractionation techniques on the immunological microenvironment was systematically examined to optimize their integration with ICI (Tojjari et al., 2024). The treatments of 3x8Gy and 18x2Gy demonstrated the most significant delay in tumor development, with the 3x8Gy regimen yielding a substantial lymphoid response, while the 18x2Gy approach facilitated a myeloid response. Granzyme B production by CD8^+^ T cells was maximized with the 3x8Gy treatment, whereas PD-L1 expression remained most consistent with 18x2Gy. The expression of TIGIT increased with the 3x8Gy regimen but decreased with the 18x2Gy approach. When combined with anti-TIGIT and anti-PD-L1 therapies, the 3x8Gy treatment achieved the highest complete response rate. These findings underscore the 3x8Gy fractionation technique as the most effective method for enhancing the efficacy of ICIs, indicating the potential of RT-ICI combinations in cancer therapy (Grapin et al., 2019).

ICIs targeting CTLA-4 and PD-1 have improved cancer immunotherapies; however, other checkpoint receptors are also identified as essential for broadening therapeutic responses (Pandey et al., 2022). PVRIG, a coinhibitory receptor belonging to the DNAM/TIGIT/CD96 nectin family that interacts with PVRL2, has been observed to diminish the production of cytokines and the cytotoxic activity of CD8^+^ T-cells (Murter et al., 2019). The antagonism between PVRIG and TIGIT, unlike CD96, has led to enhanced T-cell activity. Furthermore, it has been shown that PVRL2-mediated inhibition depends on PVRIG instead of TIGIT, thereby confirming the PVRIG/PVRL2 pathway as a distinct and nonredundant signaling axis (Murter et al., 2019). Combining PVRIG inhibition with TIGIT or PD-1 inhibitors has significantly enhanced T-cell activation. Compared to normal tissues, elevated PVRIG expression on T cells in human malignancies has been observed and linked to the expression of TIGIT and PD-1. Tumor cells coexpressing PVR and PVRL2 are common in various malignancies, particularly endometrial tumors. Expression pattern variations reveal a higher proportion of PVR^−^PVRL2^+^ cells in ovarian malignancies and PVR^−^PVRL2^+^ cells in colorectal tumors. In TILs, the blockade of PVRIG has boosted T-cell activity in specific donors, with further enhancement noted when combined with TIGIT or PD-1 inhibition. These findings suggest that the PVRIG/PVRL2 and TIGIT/PVR pathways are nonredundant and inhibitory, highlighting their importance as complementary therapeutic targets in cancer immunotherapy (Whelan et al., 2019).

In a phase Ia/Ib study, Tiragolumab demonstrated a promising safety profile in 73 patients with solid tumors, with only 4% experiencing grade 3 or higher treatment-related side effects. Fatigue and anemia were the most common adverse effects, and no dose-limiting toxicities were observed. In the metastatic NSCLC expansion cohort of 14 patients, the ORR was a remarkable 50%, and the disease control rate (DCR) was an impressive 79%, leading to a recommended dosage of 600 mg every 3 weeks (Bendell et al., 2020). The phase II CITYSCAPE study examined the combination of Tiragolumab and atezolizumab in 135 patients with advanced/metastatic PD-L1-positive NSCLC. This combination therapy enhanced ORR (37% vs. 21%) and progression-free survival (PFS; 5.6 vs. 3.9 months; hazard ratio [HR] 0.59) compared to atezolizumab alone, providing additional benefits for those with high PD-L1 expression (66% vs. 24% ORR). Some adverse events were noted, including a higher prevalence of lipase elevations in the combination therapy group, and unfortunately, two treatment-related fatalities were observed (Rodriguez-Abreu et al., 2020). These encouraging findings led to the phase III SKYSCRAPER-01 investigation. However, the interim analysis did not achieve its primary goal of PFS improvement in newly diagnosed metastatic NSCLC with PD-L1 expression ≥50% (Cho et al., 2022).

In the SCLC area, the phase III SKYSCRAPER-02 study investigated atezolizumab monotherapy or in combination with Tiragolumab alongside carboplatin and etoposide for extensive-stage illness. The interim analysis showed no significant differences in PFS (5.4 vs. 5.6 months) or overall survival (OS; 13.6 months in both groups). The ORRs and duration of response (DOR) were also very similar between the groups. The toxicity profiles were comparable, too, with roughly 70% of patients experiencing grade 3 or more significant AEs. Despite these findings, the research will continue until the final OS analysis is completed (Rudin, 2022).

The importance of immunotherapy in small cell lung cancer (SCLC) and NSCLC has been highlighted by previous trials, especially IMpower133 and CASPIAN, demonstrating the substantial benefits of combining immunotherapy with chemotherapy in extensive-stage SCLC. IMpower133 showed improved OS (12.3 vs. 10.3 months; HR 0.70) and PFS (5.2 vs. 4.3 months; HR 0.77), leading to FDA approval in 2019. Similarly, the KEYNOTE-604 study reported enhanced PFS (HR 0.75) and a higher 12-month PFS rate (13.6% vs. 3.1%) with pembrolizumab; however, OS improvements did not reach statistical significance (Rudin et al., 2020). These trials demonstrate the potential of ICIs to alter clinical outcomes while highlighting the need for further research to identify patients most likely to benefit from these treatments (Brazel et al., 2023).

A clinical trial combining the anti-TIGIT mAb Tiragolumab with atezolizumab has demonstrated improved outcomes in patients with NSCLC (Roussot et al., 2023). Preclinical evidence suggests that stereotactic body radiation therapy (SBRT) has the potential to upregulate TIGIT and PD-L1 expression, providing a strong rationale for evaluating this combination therapy. A phase I clinical trial (NCT05259319) has been designed to assess the efficacy and safety of atezolizumab, Tiragolumab, and SBRT in patients diagnosed with metastatic NSCLC, bladder cancer, renal cell carcinoma, and head and neck cancer who have previously received ICIs. The initial phase of the study focuses on metastatic NSCLC, investigating two SBRT schedules in conjunction with a fixed-dose combination of atezolizumab and Tiragolumab to ascertain the optimal administration scheme. Following this, an expansion phase will enroll additional patients with metastatic bladder cancer, renal cell carcinoma, and head and neck cancer. Participants will continue treatment until either disease progression, intolerable toxicity, or withdrawal due to intercurrent conditions or patient preference occurs. The primary endpoint of phase I is to assess the safety of the combination in sequential versus concomitant administration and establish a recommended regimen for the subsequent expansion phase. Efficacy will be evaluated in a phase II study based on a six-month PFS. Ancillary analyses will encompass assessments of peripheral and intratumoral immune biomarkers to further elucidate the immunological mechanisms underlying treatment responses (Roussot et al., 2023).

TIGIT is frequently co-expressed with PD-1 in tumor-infiltrating immune cells across various cancers, including esophageal cancer (Meyiah et al., 2023). Preliminary findings from a phase Ib dose-expansion cohort (NCT02794571) assessed the safety and efficacy of Tiragolumab in combination with atezolizumab in metastatic esophageal cancer patients who had not previously undergone cancer immunotherapy. Twenty-one patients, primarily classified as ECOG PS 1 (76.2%), with a median age of 62, were enrolled from the USA, European Union, and Asia. Most participants (71.4%) had experienced two or more prior therapies. Patients received tiragolumab (400 or 600 mg intravenously every 3 weeks) alongside atezolizumab (1,200 mg intravenously every 3 weeks) until disease progression, intolerable toxicity, or withdrawal was observed. Treatment-related adverse events (TRAEs) were reported in 66.7% of patients, with one grade 3 TRAE identified and no instances of grade 4 or 5 TRAEs recorded. Immune-mediated adverse events (imAEs) were noted in 57.1% of patients, with frequently observed adverse events comprising malignant neoplasm progression (28.6%), anemia (23.8%), decreased appetite, cough, and enzyme elevations (each 19.0%). Among the eighteen evaluable patients, the objective response rate (ORR) was documented at 27.8% (comprising five partial responses), and the DCR stood at 50%, with one patient successfully maintaining disease control for over 2 years (Wainberg et al., 2021). This combination therapy demonstrated a favorable safety profile and initial evidence of antitumor activity in a heavily pretreated population of metastatic esophageal cancer patients who had not been previously exposed to immunotherapy.

Enhanced outcomes were noted in the phase 2 CITYSCAPE trial (NCT03563716), where the combination of Tiragolumab and atezolizumab demonstrated superior results compared to monotherapy with atezolizumab (Guan et al., 2024b). The mechanism underlying the response to this combination therapy was associated with elevated baseline levels of intratumoral macrophages and Tregs, which correlated with improved outcomes in patients undergoing the combination therapy, unlike those receiving atezolizumab alone. Evaluation of serum samples revealed that macrophage activation was linked to clinical benefits in patients treated with the combined treatment. In murine tumor models, surrogate antibodies of Tiragolumab elicited inflammation in TAMs, monocytes, and DCs via Fcγ receptors (FcγR), facilitating the transition of anti-tumor CD8^+^ T cells from an exhausted effector-like state to a memory-like state. These findings elucidate a mechanism through which TIGIT checkpoint inhibitors modify immunosuppressive tumor microenvironments and underscore the importance of FcγR engagement in developing anti-TIGIT antibodies (Guan et al., 2024b).

The Phase III SKYSCRAPER-02 study aimed to evaluate whether adding Tiragolumab to atezolizumab plus carboplatin and etoposide (CE) would lead to improved outcomes for patients with untreated extensive-stage SCLC (ES-SCLC) (Rudin et al., 2024). Final analyses focused on PFS and OS. A total of 490 patients were randomized to receive either Tiragolumab or a placebo, combined with atezolizumab and chemotherapy, followed by maintenance therapy involving Tiragolumab or placebo in combination with atezolizumab. The final analysis within the primary analysis set (PAS), which comprised patients without a history or presence of brain metastases (n = 397), revealed no statistically significant improvement in PFS (stratified HR, 1.11; P = 0.3504; median PFS: 5.4 months with Tiragolumab compared to 5.6 months with the control group). The median OS in the PAS was 13.1 months for both treatment arms (stratified HR, 1.14; P = 0.2859). The results from the full analysis set (FAS), encompassing all patients regardless of brain metastases status, were consistent with the findings of the PAS. Immune-related adverse events (IrAEs) were reported in 54.4% of patients in the Tiragolumab arm and 49.2% in the control arm, respectively, with grade 3/4 events documented at 7.9% and 7.7%. Treatment withdrawal due to AEs was observed in 8.4% of patients receiving Tiragolumab and 9.3% of patients in the control group. Although no additional benefit was identified with Tiragolumab, the combination treatment was well tolerated and did not raise any new safety concerns (Rudin et al., 2024).

Tiragolumab was assessed for PK, safety, and preliminary efficacy in combination with atezolizumab in Chinese patients with advanced solid tumors during the phase I YP42514 study (Shemesh et al., 2024). A total of 20 patients received the combination therapy, with PK exposures for both agents found to be comparable to those observed in the global GO30103 study. TRAEs were consistent between Chinese and global populations, and two patients (10.0%) achieved a partial response. The combination demonstrated tolerability and preliminary anti-tumor activity, with no meaningful differences in PK or safety profiles between the populations studied (Shemesh et al., 2024).

Checkpoint inhibition with PD-L1 blockade after chemoradiotherapy (CRT) improved survival in unresectable stage III NSCLC, as shown in the PACIFIC trial, with median PFS (mPFS) of 16.9 months versus 5.6 months and median overall survival (mOS) of 47.5 months versus 29.1 months. However, 5-year overall survival remains below 50%, and eligibility for this approach is restricted to patients completing CRT without progression and with good performance status. Neoadjuvant checkpoint inhibition may increase patient eligibility, reduce tumor-related immunosuppression, and enhance tumor immunogenicity through Treg depletion and effector T-cell expansion. In the phase II AFT-16 trial, neoadjuvant atezolizumab demonstrated favorable outcomes compared to PACIFIC, with mPFS of 30 months and mOS not reached. The AFT-57 trial is a phase II randomized study designed to evaluate the safety and efficacy of neoadjuvant atezolizumab with or without Tiragolumab before and after CRT. The trial aims to identify the most effective regimen, with PFS as the primary endpoint. A total of 158 patients with stage III NSCLC, performance status 0–1, and no active autoimmune disease or organ dysfunction will be randomized to receive two cycles of atezolizumab (1,200 mg IV every 21 days) with or without Tiragolumab (600 mg IV). Non-progressing patients will undergo CRT with carboplatin, paclitaxel, atezolizumab, and thoracic radiation, followed by maintenance therapy with atezolizumab with or without Tiragolumab for up to 1 year. A pilot cohort of 20 patients will evaluate the combination of Tiragolumab and atezolizumab during CRT. Correlative studies will explore immune-related tissue and blood biomarkers to assess predictive factors. The trial, activated on 7 December 2023, began screening patients on 19 January 2024 (NCT05798663) (Ross et al., 2024).

Tiragolumab was evaluated in combination with atezolizumab in a phase I study involving Japanese patients with advanced or metastatic solid tumors (jRCT2080224926) (Yamamoto et al., 2024). Three patients with NSCLC, pancreatic cancer, and cholangiocarcinoma received Tiragolumab (600 mg) and atezolizumab (1,200 mg) intravenously every 21 days. No dose-limiting toxicities were reported, and TRAEs of any grade occurred in two patients, with no grade ≥3 AEs, serious AEs, or AEs leading to discontinuation, modification, or death. The PK parameters of Tiragolumab in cycle 1 showed a mean C_max_ of 217 μg/mL, C_min_ of 54.9 μg/mL, an area under the concentration-time curve of 2000 μg·day/mL, and a half-life of 17.6 days. The best overall response was stable disease in two patients. The combination of Tiragolumab and atezolizumab was well tolerated, and the PK profile of Tiragolumab in Japanese patients was consistent with data from non-Japanese patients in a global phase Ia/Ib study. These findings support the inclusion of Japanese patients in ongoing global phase III clinical trials (Yamamoto et al., 2024).

The SKYSCRAPER-04 phase II trial evaluated the efficacy and safety of Tiragolumab combined with atezolizumab as second or third-line therapy in patients with PD-L1-positive persistent or recurrent cervical cancer (Salani et al., 2024). Patients with PD-L1 tumor area positivity ≥5% who had received 1–2 prior chemotherapy regimens (including at least one platinum-based) were randomized in a 3:1 ratio to receive atezolizumab 1,200 mg with or without Tiragolumab 600 mg every 3 weeks until disease progression or unacceptable toxicity. The primary endpoint was the confirmed ORR by independent review per RECIST v1.1, requiring an ORR of ≥21% for statistical significance. In the Tiragolumab + atezolizumab arm (n = 126), the ORR was 19.0% (95% CI 12.6–27.0), which did not achieve statistical significance (p = 0.0787) compared to the historical reference. In the atezolizumab-alone arm (n = 45), the ORR was 15.6% (95% CI 6.5–29.5). Responses were higher in the PD-L1 high subgroup (tumor area positivity ≥10%) compared to the PD-L1 low (5%–9%) in both treatment arms. The median progression-free survival (PFS) was 2.8 months (95% CI 1.7–4.1) with the combination versus 1.9 months (95% CI 1.5–3.0) with atezolizumab alone. Post hoc analysis revealed a median OS of 11.1 months (95% CI 9.6–14.5) with the combination versus 10.6 months (95% CI 6.9–13.8) with atezolizumab. Treatment discontinuation due to adverse events occurred in 3% of the combination arm and 4% of the single-agent arm, while grade ≥3 adverse events of special interest were reported in 8% and 11%, respectively. No treatment-related deaths or new safety signals were observed. The combination of Tiragolumab and atezolizumab demonstrated a higher ORR than the historical reference but did not reach statistical significance. The regimen was well tolerated, with no new safety concerns (Salani et al., 2024).

CITYSCAPE (NCT03563716), the first randomized phase II trial of an anti-TIGIT antibody, evaluated Tiragolumab (T) combined with atezolizumab (A) (TA) versus atezolizumab alone (PA) in chemotherapy-naïve patients with PD-L1-positive (TPS ≥1%) metastatic NSCLC (Cho et al., 2021). Updated analyses of PFS, OS, and patient-reported outcomes (PROs) were presented. Eligible patients received TA (T 600 mg + A 1200 mg) or PA (A 1200 mg) intravenously every 3 weeks until disease progression or loss of clinical benefit. Co-primary endpoints included investigator-assessed ORR and PFS, while secondary endpoints included DOR, OS, safety, and PROs. Tumor PD-L1 expression levels were analyzed in relation to clinical outcomes. With a median follow-up of 16.3 months (range 0.2–35.5) in the intention-to-treat population, TA demonstrated improvements in ORR, PFS, and OS compared to PA. TRAEs occurred in 82.1% of patients receiving TA and 70.6% in the PA group, with grade 3–4 TRAEs reported in 22.4% and 25.0%, respectively. Discontinuation due to adverse events occurred in 14.9% of TA-treated patients and 13.2% of those receiving PA. Global health status and functional scale scores were maintained and comparable between arms. Clinically meaningful improvements in dyspnea (−10.6) and pain (−12.1) were observed in patients on TA who reached cycle 16. The combination of TA provided a clinically meaningful benefit, particularly in patients with PD-L1 TPS ≥50%, and maintained a manageable safety profile consistent with PD-L1/PD-1 inhibitors. Improvements in symptoms such as dyspnea and pain, along with durable responses and encouraging OS, support further evaluation of TA in metastatic PD-L1-high NSCLC (Cho et al., 2021).

Non-Hodgkin lymphoma (NHL), including follicular lymphoma (FL) and diffuse large B-cell lymphoma (DLBCL), often relapses despite current treatments, necessitating novel therapies (Chao, 2013). The immune checkpoint TIGIT, which is highly expressed on T and NK cells in NHL, was targeted in a phase Ia/Ib trial (NCT04045028) evaluating Tiragolumab, either alone or in combination with rituximab, for relapsed/refractory NHL. Among 14 patients, biomarker analysis revealed increased PD-L1 expression on immune subsets with both treatments, modest NK cell activation, and stable T-cell counts. One patient with FL exhibited a partial response lasting 11 months, correlating with enhanced NK/NKT activation and favorable TIGIT and exhaustion marker profiles. These results support tiragolumab’s potential in biomarker-driven strategies for NHL therapy, particularly in combination with PD-L1/PD-1 inhibitors (Ruppert et al., 2021).

Anti-PD-1 and anti-PD-L1 antibodies, whether alone or combined with chemotherapy or anti-CTLA-4 antibodies, are standard treatments for advanced NSCLC. Pembrolizumab or atezolizumab monotherapy significantly benefits patients with high PD-L1 expression (≥50%). However, although combinations with chemotherapy enhance overall survival (OS), they also increase adverse events (AEs). Moreover, combinations such as nivolumab plus ipilimumab demonstrate limited additional benefit (Recondo and Mezquita, 2022). TIGIT, linked to PD-1 resistance, represents a novel target for immunotherapy. The phase II CITYSCAPE trial (NCT03563716) demonstrated that the combination of Tiragolumab and atezolizumab improved ORR (38.8% vs. 20.6%) and PFS (5.6 vs. 3.9 months) compared to atezolizumab alone, particularly in patients with high PD-L1 expression (≥50%), where outcomes were markedly better. Secondary endpoints and safety were also favorable; however, the efficacy in the placebo group was lower than historical controls, possibly skewing the perceived benefit. Nonetheless, phase III SKYSCRAPER-01 data showed that the combination did not meet its PFS endpoint, leaving overall survival outcomes pending. Further studies are needed to confirm the benefits. Refine patient selection through biomarkers and exploring the potential to replace upfront chemotherapy in NSCLC treatment (Recondo and Mezquita, 2022).

The SKYSCRAPER-02C Phase III study evaluated the addition of Tiragolumab to atezolizumab and chemotherapy (carboplatin + etoposide, CE) in Chinese patients with ES-SCLC, building on prior findings from the global SKYSCRAPER-02 study, which indicated no survival benefit in a broader population (Lu et al., 2024). In SKYSCRAPER-02C, 123 patients (61 receiving Tiragolumab and 62 receiving placebo, both with atezolizumab + CE) were randomized, with 110 having no brain metastases at baseline. Results indicated numerical improvements in PFS and OS with Tiragolumab, though the study was not powered for statistical significance. The ORR was higher in the Tiragolumab arm, with a similar DOR between arms. Despite the lack of definitive survival benefit, the combination was well tolerated with no new safety concerns, supporting further exploration of Tiragolumab in ES-SCLC (Lu et al., 2024).

A study elucidated the structural basis of TIGIT inhibition by anti-TIGIT mAbs Ociperlimab and Tiragolumab, blocking the interaction between TIGIT and its ligand, PVR, in the TME (Sun et al., 2024b). Crystal structures of TIGIT bound to the Fab fragments of Ociperlimab and Tiragolumab reveal that both mAbs induce significant steric hindrance with PVR and interact with key epitopic residues critical for their inhibitory function. Interestingly, Ociperlimab exhibits a 17-fold increase in binding affinity to TIGIT under acidic conditions (pH 6.0) compared to neutral pH (7.4), which is attributed to a strong electrostatic interaction between ASP103 in its heavy-chain CDR3 and HIS76 on TIGIT. In contrast, Tiragolumab does not demonstrate pH-dependent binding enhancement. These findings provide molecular insights that may inform the design and optimization of more effective TIGIT-targeting therapeutic antibodies for cancer immunotherapy (Sun et al., 2024b).

Tiragolumab also enhanced efficacy when combined with atezolizumab in the phase 2 CITYSCAPE trial (Guan et al., 2024a). Improved outcomes were associated with elevated baseline levels of intratumoral macrophages and Treg, correlating with clinical benefit in the combination treatment group but not with atezolizumab monotherapy. Serum analysis revealed that macrophage activation occurred during treatment and was linked to favorable outcomes. In syngeneic mouse tumor models, Tiragolumab surrogate antibodies activated TAMs, monocytes, and DCs via FCγR engagement, leading to a transition of CD8^+^ T cells from an exhausted effector-like phenotype to a memory-like state. These findings highlight Tiragolumab’s capacity to modulate the immunosuppressive TME and the critical role of FCγR interactions in the therapeutic mechanism of anti-TIGIT antibodies (Guan et al., 2024a).

Microsatellite-stable colorectal cancers (MSS-CRC) demonstrated resistance to anti-PD-1/PD-L1 therapies, but *ex vivo* analyses indicated that combining atezolizumab with Tiragolumab partially restored immune reactivity in TILs (Thibaudin et al., 2022). Among 13 MSS-CRC and 3 microsatellite instable (MSI) tumors analyzed, atezolizumab alone reactivated T cells only in MSI tumors, whereas the combination therapy reactivated T cells in 46% of MSS-CRC samples. Reactivation was associated with higher baseline frequencies of Th1 and Tc1 cells, elevated T cell polyfunctionality, and increased CD96 expression. Bioinformatic analyses of public single-cell RNA sequencing and TCGA datasets supported these findings, identifying CD96 expression as a potential predictive marker for the efficacy of this combination. These results suggested that atezolizumab and Tiragolumab may restore CD4^+^ and CD8^+^ TIL function in MSS-CRC, warranting further clinical evaluation in this patient population (Thibaudin et al., 2022).

Emerging evidence highlights the synergistic potential of combining anti-TIGIT antibodies with LAG-3 or CTLA-4 inhibitors in cancer immunotherapy. Preclinical studies demonstrate that TIGIT, LAG-3, and CTLA-4 contribute to T-cell exhaustion through distinct mechanisms, and their co-blockade enhances CD8^+^ T-cell responses, reduces Treg-mediated immunosuppression, and delays tumor growth in murine models (Qin et al., 2019; Huang et al., 2017). For instance, dual blockade of TIGIT and LAG-3 in ovarian cancer models overcame compensatory checkpoint upregulation, while a tri-specific PD-L1/TIGIT/LAG-3 antibody outperformed benchmark therapies in T-cell expansion and tumor suppression (Huang et al., 2017; Yang et al., 2023). Similarly, TIGIT blockade synergizes with CTLA-4 inhibition by restoring CD226 co-stimulatory signaling, improving progression-free survival in melanoma models (Ge et al., 2021). Clinically, the FDA approval of a LAG-3/TIGIT bispecific antibody (BsAb) for advanced solid tumors and promising results from trials like CITYSCAPE (tiragolumab plus atezolizumab in NSCLC) underscore the therapeutic potential of these combinations, though CTLA-4-based triple therapies remain in early-phase evaluation (Rousseau et al., 2023; Zhang et al., 2024). These findings suggest that multi-checkpoint blockade targeting TIGIT with LAG-3 or CTLA-4 could address resistance to single-agent therapies, warranting further investigation in large-scale clinical trials.

A multivalent BsAb was developed to co-target PD-L1 and TIGIT, combining a tetravalent anti-PD-L1 Fc-fusion nanobody (Nb) with a tetravalent anti-TIGIT Nb (Ma et al., 2020). The parental anti-PD-L1 Nb demonstrated high specificity and affinity for primate PD-L1, enhanced T cell activity *in vitro*, and effective anti-tumor activity *in vivo*. Similarly, the anti-TIGIT Nb exhibited high specificity and affinity for primate TIGIT with comparable immune-activating effects. The BsAb maintained significant blocking activity against PD-1/PD-L1 and TIGIT/CD155 interactions and synergistically enhanced T cell activity *in vitro* compared to the individual Nbs. These findings underscore the potential of the BsAb for further *in vivo* studies as a candidate for improving anti-tumor immune responses (Ma et al., 2020).

**FIGURE 3:**
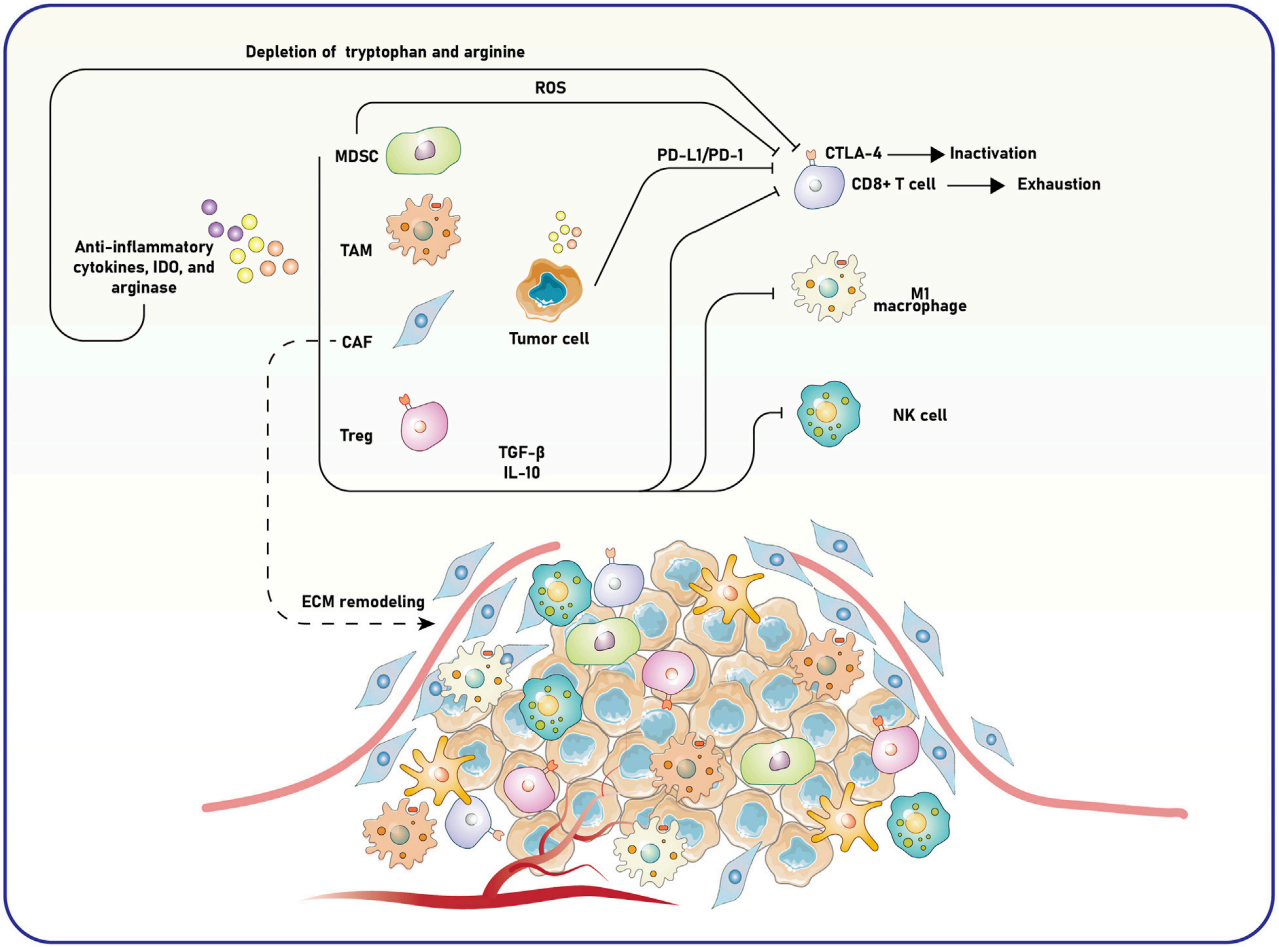
Combination strategies targeting TIGIT.

The studies reviewed highlight the significant potential of TIGIT as a therapeutic target in cancer immunotherapy (Table 1). Evidence derived from preclinical and clinical trials underscores its ability to enhance anti-tumor immune responses, particularly when used with other immunotherapeutic agents, such as PD-1/PD-L1 inhibitors. Despite the encouraging results observed in specific patient subsets, especially those exhibiting elevated PD-L1 expression, persistent challenges hinder the consistent attainment of statistically significant survival benefits across broader populations. Ongoing investigations into combination therapies, biomarkers, and innovative mechanisms of action present promising avenues for optimizing TIGIT-based strategies and improving clinical outcomes across various cancers (Figure 3).

**TABLE 1:** Using anti-TIGIT mAb alone and with other anti-cancer agents.

Intervention	Type of study	Mechanism of action	Outcomes	References
TIGIT Blockade	Preclinical study in tumor-bearing mice	Inhibits NK cell depletion; promotes NK/T-cell-mediated tumor immunity	Enhanced tumor-specific immunity, synergistic effects with PD-L1 blockade, durable memory immunity	Zhang et al. (2018)
Tiragolumab + Atezolizumab	Phase Ia/Ib GO30103 trial	Tiragolumab modulates immune checkpoints; exhibits biphasic PK profile	Safe with no dose-limiting toxicities, effective antitumor activity, ORR 46% (NSCLC), 28% (esophageal cancer)	Kim et al. (2023), Garralda et al. (2024)
Radiotherapy + Anti-TIGIT/PD-L1	Preclinical study	Maximizes Granzyme B production; modifies immune response	High complete response rates in RT-ICI combinations with 3x8Gy fractionation	Grapin et al. (2019)
PVRIG + TIGIT/PD-1 Inhibitors	Exploratory study	Amplifies T-cell activation by blocking nonredundant pathways	Enhanced T-cell activity in TILs and *in vitro* assays	Whelan et al. (2019)
Tiragolumab (Safety Profile)	Phase Ia/Ib trial	Monoclonal antibody targeting TIGIT	ORR of 50% in metastatic NSCLC; DCR of 79%; well-tolerated	Bendell et al. (2020)
Tiragolumab + Atezolizumab	Phase II CITYSCAPE trial	TIGIT inhibition leads to FcγR-mediated macrophage and T-cell activation	Improved ORR (37% vs. 21%), longer PFS (5.6 vs. 3.9 months), especially in PD-L1-high NSCLC patients	Rodriguez-Abreu et al. (2020)
Tiragolumab + Chemotherapy	Phase III SKYSCRAPER-02 study	Combined checkpoint inhibition and chemotherapy	No significant differences in PFS or OS in extensive-stage SCLC patients	Rudin (2022), Lu et al. (2024)
Pembrolizumab (KEYNOTE-604 Study)	Phase III study	Anti-PD-1 therapy	Improved PFS (HR 0.75); higher 12-month PFS rate; OS not statistically significant	Rudin et al. (2020)
Tiragolumab + SBRT	Phase I study	Enhances TIL function and antitumor response	Safety and efficacy of sequential/concomitant administration under investigation	Roussot et al. (2023)
Tiragolumab in Esophageal Cancer	Phase Ib dose-expansion cohort	Blocks TIGIT/CD155 interaction	ORR 27.8%, DCR 50%, well-tolerated	Wainberg et al. (2021)
Tiragolumab in CITYSCAPE Analysis	Phase II trial (updated analysis)	FcγR engagement enhances macrophage and T-cell activation	Clinically meaningful improvements in symptoms and durable responses	Guan et al. (2024b), Guan et al. (2024a)
Tiragolumab + Atezolizumab	Phase III SKYSCRAPER-01 study	Immune checkpoint inhibition targeting TIGIT	Did not meet PFS endpoint; OS outcomes pending	Rudin et al. (2024), Recondo and Mezquita (2022)
Tiragolumab + Atezolizumab in China	Phase I YP42514 study	TIGIT inhibition and FcγR engagement	Well-tolerated, consistent PK/safety profiles with global studies	Shemesh et al. (2024)
Tiragolumab in Japanese Patients	Phase I study (jRCT2080224926)	Consistent PK parameters and tolerability across populations	Stable disease observed in 2 patients	Yamamoto et al. (2024)
Tiragolumab in Cervical Cancer	Phase II SKYSCRAPER-04 trial	TIGIT inhibition in PD-L1-positive tumors	Did not reach statistical significance for ORR; well-tolerated safety profile	Salani et al. (2024)
CITYSCAPE (Detailed Analysis)	Phase II CITYSCAPE trial (PD-L1-high NSCLC)	Enhanced macrophage and T-cell activation, FcγR engagement	Durable responses, encouraging OS, and symptom improvements in PD-L1-high patients	Cho et al. (2021)
Tiragolumab in NHL	Phase Ia/Ib study	Targets TIGIT to activate NK cells and stabilize T-cell counts	Partial response in 1 patient; supports biomarker-driven strategies	Ruppert et al. (2021)
TIGIT in MSS-CRC	*Ex vivo* analysis + bioinformatics study	Restores immune reactivity in TILs	Reactivated T-cells in 46% of MSS-CRC samples; CD96 identified as predictive marker	Thibaudin et al. (2022)
Anti-TIGIT Bispecific Antibody	Preclinical study	Blocks PD-1/PD-L1 and TIGIT/CD155 pathways synergistically	Enhanced T-cell activity *in vitro*, potent anti-tumor immune responses	Ma et al. (2020)
Structural Insights into TIGIT mAbs	Structural analysis of Ociperlimab + Tiragolumab	Steric hindrance with PVR; ligand-specific blocking	Key epitopic residues identified; potential for optimized TIGIT-targeting therapies	Sun et al. (2024b)

## 5 Challenges and limitations

**FIGURE 4:**
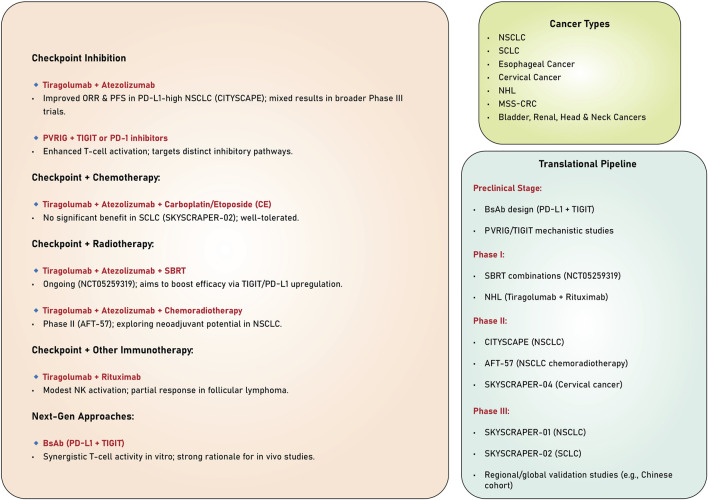
Challenges and opportunities of TIGIT blockade.

Despite the initial promise of anti-TIGIT therapies, such as Tiragolumab, their clinical application has uncovered significant challenges that dampen enthusiasm for this approach in cancer immunotherapy (Nguyen et al., 2024; Socinski, 2024). These hurdles—arising from biological complexity, variable clinical outcomes, and therapeutic resistance—necessitate further investigation to optimize TIGIT blockade. However, they also provide opportunities to explore alternative strategies and develop more comprehensive, holistic models in precision medicine to enhance therapeutic success (Figure 4).

### 5.1 Biological challenges

TIGIT’s expression across diverse immune cell types—including CD8^+^ T cells, Tregs, and NK cells—creates a complex therapeutic landscape (Ge et al., 2021). While TIGIT blockade aims to reinvigorate exhausted effector T cells to boost anti-tumor immunity, its presence on Tregs and NK cells introduces competing effects (Wu et al., 2019). Anti-TIGIT antibodies with Fc-active domains, such as Tiragolumab, can trigger ADCC, potentially depleting TIGIT^+^ Tregs or NK cells (Hasan et al., 2023). This “fratricide” may undermine efficacy by impairing NK cell-mediated tumor killing or disrupting Treg-mediated immune homeostasis, particularly in NK cell-dependent tumor contexts (Hasan, 2023; Hasan et al., 2023). To address this, next-generation anti-TIGIT antibodies with Fc-inert domains are being developed to prevent ADCC-mediated depletion, preserving the beneficial roles of Tregs and NK cells. Additionally, BsAbs targeting TIGIT alongside other checkpoints (e.g., PD-1 or LAG-3) offer a promising alternative to enhance specificity and minimize off-target effects. For instance, BiPT-23, an IgG1-type BsAb targeting PD-L1 and TIGIT, has shown preclinical promise by selectively depleting TIGIT^+^ Tregs while maintaining other immune cells in the TME (Zhong et al., 2022). Similarly, ZGGS15, an IgG4 BsAb targeting LAG-3 and TIGIT, demonstrated potent anti-tumor efficacy without inducing ADCC, suggesting a safer and more effective approach (Dai et al., 2024).

### 5.2 Clinical challenges

Tiragolumab initially showed encouraging results in preclinical studies and early-phase trials, particularly in combination with anti-PD-L1 agents like atezolizumab (Chu et al., 2023). However, larger trials, such as those in non-small cell lung cancer (NSCLC), have failed to consistently demonstrate significant survival benefits (Cai et al., 2023). Moreover, severe irAEs, such as immune-mediated hepatitis, have emerged as critical concerns. A notable case involved a 64-year-old man with laryngeal squamous cell carcinoma who developed liver failure after receiving atezolizumab and Tiragolumab, though he recovered following plasma exchange therapy (Renault et al., 2023). These mixed outcomes highlight the need for improved patient selection and toxicity management. Unlike PD-1/PD-L1 inhibitors, which rely on PD-L1 expression as a biomarker, anti-TIGIT therapies lack validated predictive markers (Chen et al., 2020; Shen et al., 2019; Villaruz et al., 2019; Yu et al., 2016). Ongoing research using advanced techniques like single-cell RNA sequencing aims to identify biomarkers, such as TIGIT, CD96, or DNAM-1 expression levels, that could effectively stratify patients (Stamm et al., 2018; Shibru et al., 2021; Saha, 2023). Additionally, early intervention strategies, such as plasma exchange for irAEs, could enhance safety and tolerability in clinical settings.

### 5.3 Resistance and pharmacokinetic challenges

Therapeutic resistance to TIGIT blockade can stem from tumor-intrinsic factors, such as low immunogenicity or poor antigen presentation, which limit T cell activation despite TIGIT inhibition (Kalbasi and Ribas, 2020). Furthermore, upregulation of alternative immune checkpoints, like TIM-3 or LAG-3, may compensate for TIGIT blockade, reducing its efficacy (Borgeaud et al., 2023). To counter these challenges, combination therapies offer a viable solution. Agents that enhance tumor immunogenicity, such as oncolytic viruses or cancer vaccines, could synergize with TIGIT inhibitors to improve immune activation. Co-targeting additional checkpoints, such as LAG-3 or TIM-3, may also prevent compensatory resistance, with preclinical evidence supporting the synergy of TIGIT and LAG-3 blockade (Cai et al., 2023; Lu and Tan, 2024). These approaches aim to create a more permissive TME for anti-TIGIT therapy, addressing resistance at multiple levels. Achieving sufficient receptor occupancy (RO) within the TME remains a pharmacokinetic hurdle, as high peripheral RO does not always correlate with intratumoral levels (Desai and Subbiah, 2023; Piovesan et al., 2024). This discrepancy may explain some of Tiragolumab’s inconsistent clinical results. Physiologically based pharmacokinetic (PBPK) modeling has been proposed to optimize dosing regimens, predicting nearly complete RO in peripheral blood and tumors for antibodies like ociperlimab and vibostolimab (Shchelokov et al., 2022). Such models could guide dose adjustments to maximize intratumoral efficacy. Additionally, alternative delivery methods, such as intratumoral injections, may increase local drug concentrations, offering a practical solution to enhance therapeutic impact (Garralda et al., 2024; Tang, 2021).

The limitations of anti-TIGIT therapies, exemplified by Tiragolumab, reflect the intricate interplay of biological, clinical, and pharmacological factors in cancer immunotherapy. While these challenges highlight gaps in our current approach, they also point to actionable solutions—Fc-inert or BsAbs, biomarker-driven patient selection, combination regimens, and advanced pharmacokinetic strategies. Ultimately, realizing the full potential of TIGIT blockade requires robust, holistic models in precision medicine that integrate these elements. By tailoring therapies to individual tumor and immune profiles, such models can bridge the gap between critique and innovation, paving the way for more effective and personalized cancer treatments.

## 6 Future perspectives

Exploring TIGIT as an ICI has revealed its critical function in immune evasion within the TME. Tiragolumab, a mAb specifically targeting TIGIT, has demonstrated efficacy in preclinical and early clinical trials, mainly when combined with anti-PD-L1 agents. Despite its promise, several challenges persist, including resistance mechanisms, the absence of robust biomarkers, and inconsistent outcomes in phase III trials. These challenges emphasize the necessity for innovative strategies to optimize TIGIT-targeted therapies. The application of BsAbs presents a promising avenue for advancing cancer immunotherapy. For instance, BiPT-23, an IgG1-type BsAb targeting PD-L1 and TIGIT, has shown remarkable potential by enhancing cytotoxic T cell and NK cell infiltration while selectively depleting TIGIT^+^ Tregs (Zhong et al., 2022). Similarly, ZGGS15, a bispecific IgG4 antibody targeting LAG-3 and TIGIT, has demonstrated superior antitumor activity and synergy with nivolumab, achieving significant tumor growth inhibition without inducing adverse immunological effects (Dai et al., 2024). Lastly, developing HLX53, a single-domain antibody targeting TIGIT, has underscored the advantages of innovative formats in augmenting tumor penetration and immune activation, particularly when combined with anti-PD-L1 therapies (Hua et al., 2021). Integrating BsAb technologies within TIGIT-targeted strategies represents a significant advancement in immunotherapy. Future research endeavors should prioritize optimizing BsAb formats to establish a balance between efficacy and safety. Furthermore, it is essential to identify biomarkers that enable the stratification of patient populations expected to benefit from these therapies and clarify the mechanisms underlying resistance to TIGIT blockade. Additionally, investigating combinatorial regimens incorporating complementary checkpoint inhibitors or radiation therapy can significantly enhance therapeutic outcomes. When paired with advancements in pharmacokinetics and dosing optimization, these strategies may address current limitations and fully leverage the inherent potential of TIGIT-targeted therapies in oncology.

## 7 Concluding remarks

The exploration of tiragolumab, a monoclonal antibody targeting TIGIT, represents a significant advancement in cancer immunotherapy, particularly within the context of the immunosuppressive TME. Preclinical and early clinical trials, such as the phase II CITYSCAPE trial, have demonstrated that tiragolumab, especially in combination with anti-PD-L1 agents like atezolizumab, enhances anti-tumor immunity by reinvigorating CD8^+^ T cells and NK cells, leading to improved objective response rates and progression-free survival in cancers like NSCLC and esophageal cancer. These findings underscore TIGIT’s critical role in immune evasion and highlight the potential of its blockade to restore immune effector functions. However, phase III trials, including SKYSCRAPER-01 and SKYSCRAPER-02, have revealed challenges, with no significant survival benefits observed in NSCLC and small cell lung cancer, suggesting limitations due to tumor heterogeneity, compensatory immune checkpoints (e.g., TIM-3, LAG-3), and inadequate patient selection criteria. The development of BsAbs, such as BiPT-23 and ZGGS15, and novel formats like HLX53, offers promising strategies to overcome these hurdles by enhancing immune cell infiltration and synergy with existing therapies. Optimizing therapies that target TIGIT necessitates the development of robust biomarkers, the refinement of trial designs, and a profound understanding of resistance mechanisms in order to fully harness their therapeutic potential in personalized cancer treatment.
